# Supplementary material for: Contrasting patterns of water use efficiency and annual radial growth among European beech forests along the Italian peninsula
Source: Sci Rep. 2024 Mar 19;14:6526. doi: 10.1038/s41598-024-57293-7 (PMC11350120; doi:10.1038/s41598-024-57293-7)
Supplement: Supplementary file 1 — Supplementary Information. [file 41598_2024_57293_MOESM1_ESM.docx]

**Supplementary information**

**Contrasting patterns of water use efficiency and annual radial growth among European beech forests along the Italian peninsula**

**Supplementary Table 1** Soil type and the percentage composition of clay, silt, sand and soil water holding capacity (SWHC) at one meter depth for each site.

| Site | Soil type | Clay (%) | Silt (%) | Sand (%) | Soil water holding capacity (SWHC) |
| --- | --- | --- | --- | --- | --- |
| TRE | Udivitrands, Andisols | 19.9 | 37.8 | 42.3 | Low |
| LAZ | Haploxeralfs, Luvisols | 30.5 | 41.1 | 28.3ssssss | High |
| CAM | Dystrudepts, Inceptisols | 30.8 | 37.8 | 31.4 | High |
| CAL | Dystrudepts, Inceptisols | 25.8 | 41.5 | 32.7 | Moderate |

**Tree-ring chronology building**

Cross-dated measurements of TRW and BAI were standardized to remove the size/age related trends using a age-dependent 50-year smoothing spline to produce standard chronology and maintain the low frequency signal. The method is effective in capturing the rapid growth of juvenile trees with a flexible spline, transitioning to a more rigid spline that accurately models the consistent growth typical of mature trees (Cook and Kairiukstis 1990; Fritts 1976, Supplementary Fig. 2).

The Expressed Population Signal (EPS) statistic was used to assess the strength of the common signal among the TRW series in a chronology over time. We considered an EPS > 0.85, indicating the dominance of the stand-level signal over individual tree signals. The mean sensitivity statistic was used to assess interannual variation among the tree rings (Fritts 1976). All analyses were restricted to the period covered by the youngest trees (at LAZ), i.e., EPS, from 1965 until 2014 (Supplementary Table 2). All computations were performed using the R package dplR (Bunn, 2008).

**Supplementary Table 2.** Dendrochronological statistics (means ± SE) of the tree-ring width series.

| Site | No. trees | First-last years | Tree-ring width (mm) | Correlation with mean series | EPS |
| --- | --- | --- | --- | --- | --- |
| TRE | 40 | 1888-2016 | 1.01 ± 0.52 | 0.53 ± 0.11 | 1935 |
| LAZ | 20 | 1942-2014 | 2.05 ± 0.81 | 0.72 ± 0.08 | 1965 |
| CAM | 65 | 1877-2016 | 0.98 ± 0.25 | 0.51 ± 0.23 | 1910 |
| CAL | 50 | 1938-2017 | 1.43 ± 0.39 | 0.64 ± 0.09 | 1960 |

**Supplementary Table 3** Tukey post-hoc test for TRW and age in the study sites (one-way analysis of variance with Tukey’s Honest Significant Difference test). Different letters indicate significant differences (*P* < 0.05).

| Sites | TRW (mm) | Tukey´s post-hoc | Age (years) | Tukey´s post-hoc |
| --- | --- | --- | --- | --- |
| TRE | 1.01 | *b* | 78 | *a* |
| LAZ | 2.05 | *a* | 53 | *b* |
| CAM | 0.98 | *c* | 102 | *c* |
| CAL | 1.43 | *b* | 63 | *d* |

**Supplementary Table 4** Statistic of the generalized additive mixed models for the basal area increment (BAI) trends of *Fagus sylvatica* for each site.

| Family | Model Formula | | | R^2^(adj) |
| --- | --- | --- | --- | --- |
| Gaussian | *BAI_i_=s[year_i_*(Site)]+s(age_i_)+s(SPEI18_i_)+Z_i_B_i_+ɛ* | | | 0.654 |
| Parametric coefficients |  |  |  |  |
|  | Estimate | Std. Error | t value | Pr(>\|t\|) |
| (Intercept) | 5.943 0.218 27.25 | | | <.0001 |
|  |  |  |  |  |
| Approximate significance of smooth terms: | | | | |
|  | edf | Ref.df | *F*-value | *P*-value |
| s(year):SiteTRE | 2.988 | 2.988 | 361.3 | <.0001 |
| s(year):SiteLAZ | 2.993 | 2.993 | 686.2 | <.0001 |
| s(year):SiteCAM | 2.595 | 2.595 | 233.4 | <.0001 |
| s(year):SiteCAL | 2.996 | 2.996 | 521.62 | <.0001 |
| s(Age) | 2.981 | 2.981 | 150.1 | <.0001 |
| s(spei18) | 2.839 | 2.839 | 32.0 | <.0001 |
| s(tree) | 298.39 | 303 | 79.9 | <.0001 |


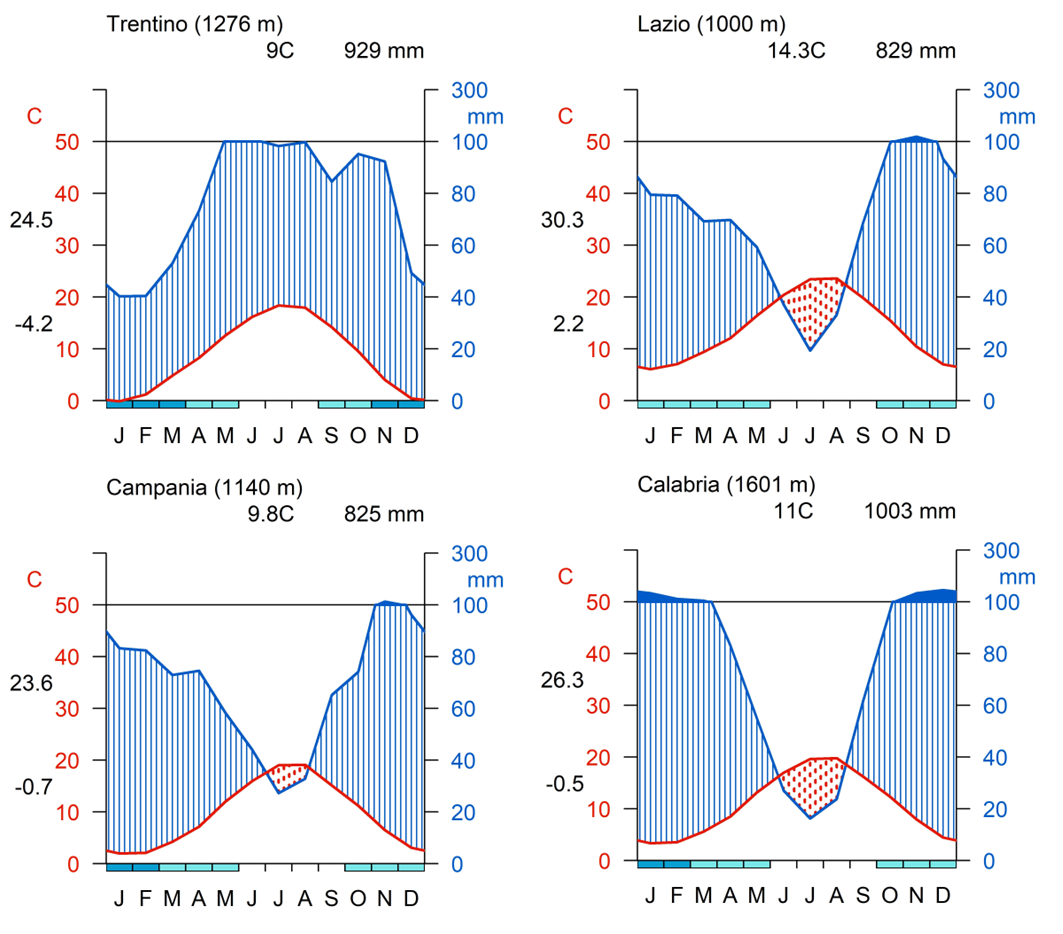


**Supplementary Fig. 1** Walter-Lieth climatograms for the four study sites: Trentino, Lazio, Campania, and Calabria (from north to south, left to right) for the period 1965-2014. The lower blue bars show the frost period.

**
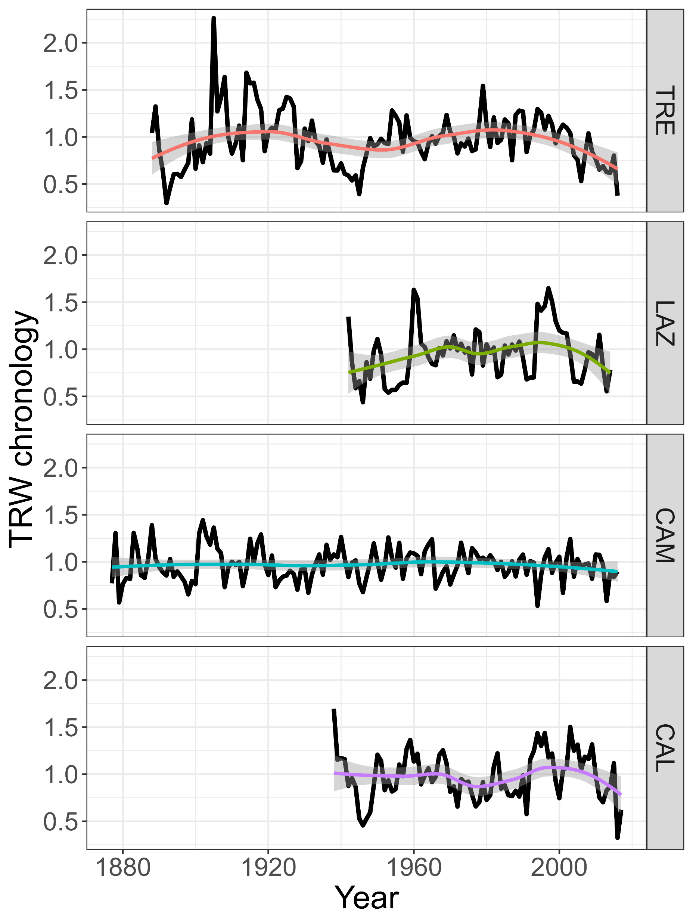
**

**Supplementary Fig. 2** Tree-ring width age dependent 50 years spline chronology of *Fagus sylvatica* for each site. Colour lines for each site indicate the nonlinear model fitted using the loess smoothing method, shaded areas represent 95 % confidence intervals.


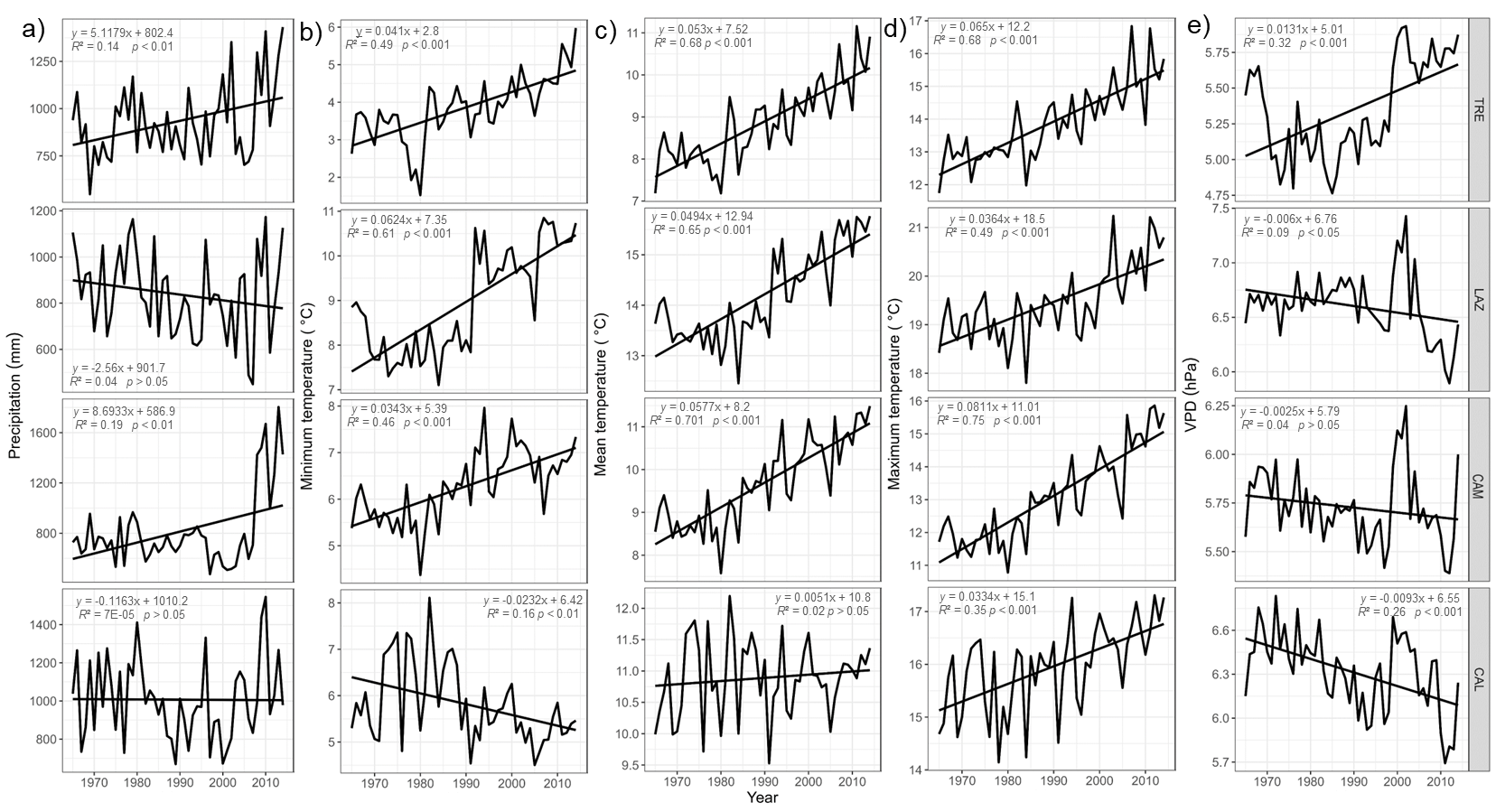
 **Supplementary Fig. 3** Climate trends at the four sites TRE, LAZ, CAM and CAL (from north to south) for the period 1965 – 2014: Mean annual a) precipitation; b) minimum temperature; c) mean temperature; d) maximum temperature and e) vapour pressure deficit (VPD). Linear regression lines are also indicated.


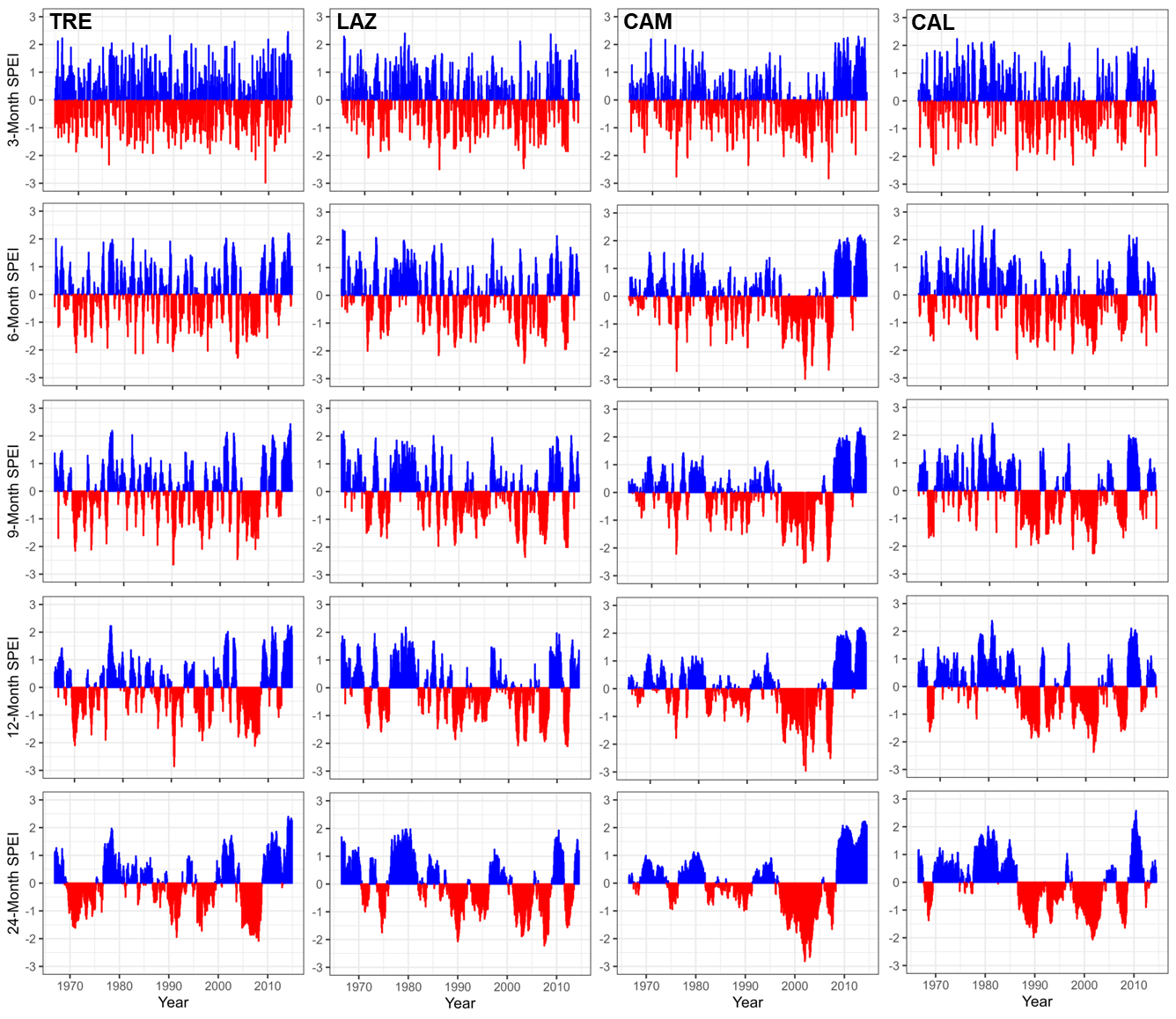


**Supplementary Fig. 4** Standardized 3-6-9-12-and 24 months SPEI at the four study sites (TRE, LAZ, CAM and CAL) for the 1965–2014 period. Negative (red) and positive (blue) values indicate drier and wetter conditions, respectively.

**Supplementary Fig. 5** Pearson’s running correlations between BAI age-dependent 50-yrs spline chronology with monthly precipitation, mean temperature, VPD, and SPEI1 for the current and the previous year (*), over the period 1965-2014 at each site. The *y-axis* represents the time window in months. Colours (see the key) represent correlation coefficients that are significant at the level of *r* = 0.279 (*P*< 0.05).


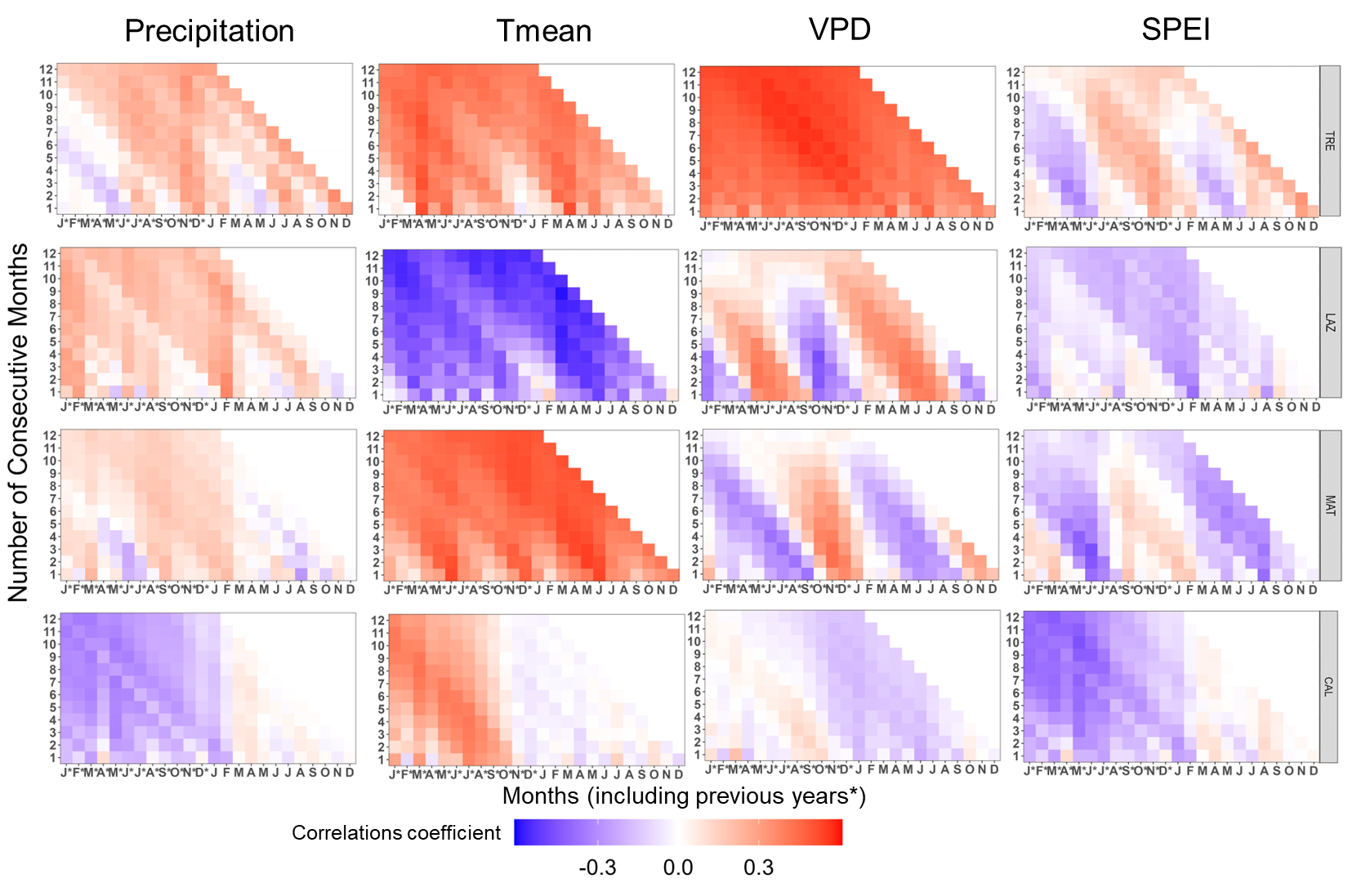


**Supplementary Fig. 6** Pearson’s running correlations between δ^13^C with monthly precipitation, mean temperature, VPD, and SPEI1 for the current and the previous year (*), over the period 1965-2014 at each site. The *y-axis* represents the time window in months. Colours (see the key) represent correlation coefficients that are significant at the level of *r* = 0.279 (*P*< 0.05).
